# Inhibition of spontaneous and experimental lung metastasis of soft-tissue sarcoma by tumor-targeting *Salmonella typhimurium* A1-R

**DOI:** 10.18632/oncotarget.2561

**Published:** 2014-12-30

**Authors:** Shinji Miwa, Yong Zhang, Kyung-Eun Baek, Fuminari Uehara, Shuya Yano, Mako Yamamoto, Yukihiko Hiroshima, Yasunori Matsumoto, Hiroaki Kimura, Katsuhiro Hayashi, Norio Yamamoto, Michael Bouvet, Hiroyuki Tsuchiya, Robert M. Hoffman, Ming Zhao

**Affiliations:** ^1^ AntiCancer, Inc., San Diego, California, USA; ^2^ Department of Surgery, University of California, San Diego, San Diego, California, USA; ^3^ Department of Orthopedic Surgery, Kanazawa University Graduate School of Medical Sciences, Kanazawa, Ishikawa, Japan

**Keywords:** HT-1080, orthotopic model, nude mice, lung metastasis, bacterial therapy

## Abstract

Prognosis of patients with lung metastases of soft-tissue sarcoma is still poor. Therefore, novel systemic therapy is needed to improve the survival of soft-tissue sarcoma. In the present study, tumor-targeting therapy with a genetically-modified auxotrophic strain of *Salmonella typhimurium*, termed A1-R, was evaluated. Mouse models of primary soft tissue sarcoma and spontaneous lung metastasis were obtained by orthotopic intra-muscular injection of HT1080-RFP human fibrosarcoma cells. *S. typhimurium* A1-R was administered from day 14, once a week for two weeks. On day 28, lung samples were excised and observed with a fluorescence imaging system. The number of lung metastasis was 8.8 ± 3.4 in the untreated group and 0.8 ± 0.8 in the treated group (*P* = 0.024). A mouse model of experimental lung metastasis was obtained by tail vein injection of HT1080-RFP cells. The mice were treated with *S. typhimurium* A1-R (i.v.) on day 7, once a week for three weeks. *S. typhimurium* A1-R significantly reduced lung metastases and improved overall survival (*P* = 0.004). *S. typhimurium* A1-R bacterial therapy has future potential for treating advanced soft tissue sarcoma and improving prognosis of patients with lung metastasis.

## INTRODUCTION

The 5-year survival rate of the patients with lung metastases from soft-tissue sarcoma is 15.5% [1]. Systemic control of soft tissue sarcoma is necessary in the treatment of this disease. Although chemotherapy is widely used as the systemic treatment for soft tissue sarcoma, it has failed to show long-term survival benefits [2]. Therefore, novel systemic therapy is needed to improve the outcome of soft tissue sarcoma.

Our laboratory developed a *Salmonella typhimurium* (*S. typhimurium*) A1-R strain that has high tumor-colonization and antitumor efficacy. *S. typhimurium* A1-R is auxotrophic for leu-arg, which prevents it from continuously infecting normal tissues. *S. typhimurium* A1-R has no other apparent attenuating mutations *S. typhimurium* A1-R could eradicate primary and metastatic tumors as monotherapy in nude mice with prostate [3,4], breast [5], lung [6,7] and pancreatic [8, 9] cancers, including pancreatic cancer stem cells [10] and pancreatic cancer patient-derived orthotopic xenografts [PDOX] [11], as well as sarcoma [12, 13] and glioma [14, 15].

Treatment with tumor-targeting *S. typhimurium* A1-R completely prevented the appearance of bone metastasis of a high metastatic variant of breast cancer in nude mice [16].

In our previous study, *S. typhimurium* A1-R was administered i.v. to nude mice which had primary osteosarcoma bone tumor and lung metastasis. The primary bone tumor developed after orthotopic intra-tibial injection of 143B-RFP (red fluorescent protein) human osteosarcoma cells. *S.typhimurium* A1-R was effective against both the primary bone tumor and lung metastasis [13].

*S. typhimurium* A1-R, expressing green fluorescent protein (GFP), was administered to nude mice with popliteal lymph node metastasis of human HT-1080 fibrosarcoma as well as lung metastasis of the fibrosarcoma. *S. typhimurium* A1-R was delivered via a lymphatic channel to target the lymph node metastases and systemically via the tail vein to target the lung metastasis. The sarcoma cells expressed RFP in the cytoplasm and GFP in the nucleus linked to histone H2B, enabling color-coded real-time imaging of the GFP-expressing bacteria targeting the metastases. After 7–21 days of treatment, the metastases were eradicated without the need of chemotherapy or any other treatment. No adverse effects were observed [12].

Intratumoral injection of *Clostridium novyi* spores with the toxin gene knocked out (*C. novyi*-NT) was administered to dogs with solid tumors. Responses were observed in 6 of 16 dogs. A human patient with advanced leiomyosarcoma was treated with an intratumoral (i.t.) injection of *C. novyi*-NT spores which reduced the tumor's size [17]. However, obligate anaerobes such as *C. novyi* or *Bifidobactum* [18] may not be appropriate for metastatic cancer since they are seemingly only active with *i.t.* administration. If bacterial therapy is going to be widely available and efficacious, it has to target metastatic cancer.

In the present study, we determined the efficacy of *S. typhimurium* A1-R on primary tumors and experimental and spontaneous metastasis in mouse models of human soft-tissue sarcoma.

## RESULTS AND DISCUSSION

### Color-coded imaging of the interaction of *S. typhimurium* A1-R-GFP with RFP-expressing HT-1080 fibrosarcoma cells

**Figure 1:**
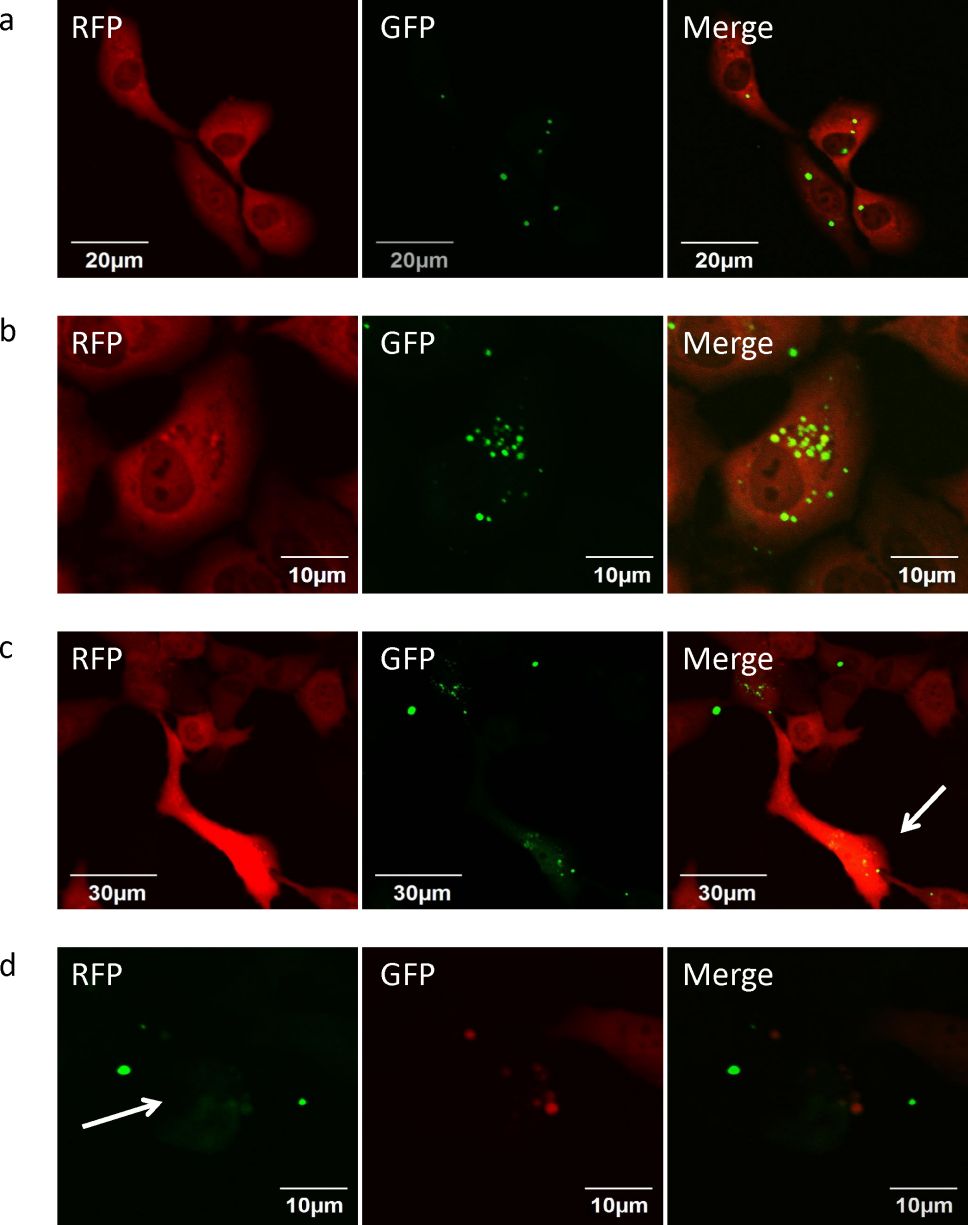
Efficacy of *S. typhimurium* A1-R on HT-1080 fibrosarcoma cells *in vitro* **(a)** Early interaction of *S. typhimurium* A1-R-GFP and HT-1080-RFP cells. **(b)** Increase of *S. typhimurium* A1-R-GFP in the cytoplasm of HT-1080-RFP cells. **(c)** Apoptosis of HT-1080-RFP cells induced by *S. typhimurium* A1-R. **(d)** Fragmentation of cytoplasm of *S. typhimurium* A1-R-GFP-treated HT-1080-RFP cells.

The interaction between *S. typhimurium* A1-R expressing GFP and HT-1080 fibrosarcoma cells labeled with RFP was observed with the Fluoview FV1000 confocal microscope (Olympus Corp., Tokyo, Japan). GFP-expressing *S. typhimurium* A1-R invaded the fibrosarcoma cells (Fig. 1a) and proliferated in the cytoplasm (Fig. 1b). The proliferation of *S. typhimurium* A1-R in the cytoplasm of fibrosarcoma cells induced cell death (Fig. 1c, d).

### Specific targeting of *S. typhimurium* A1-R to soft tissue sarcoma *in vivo*

**Figure 2:**
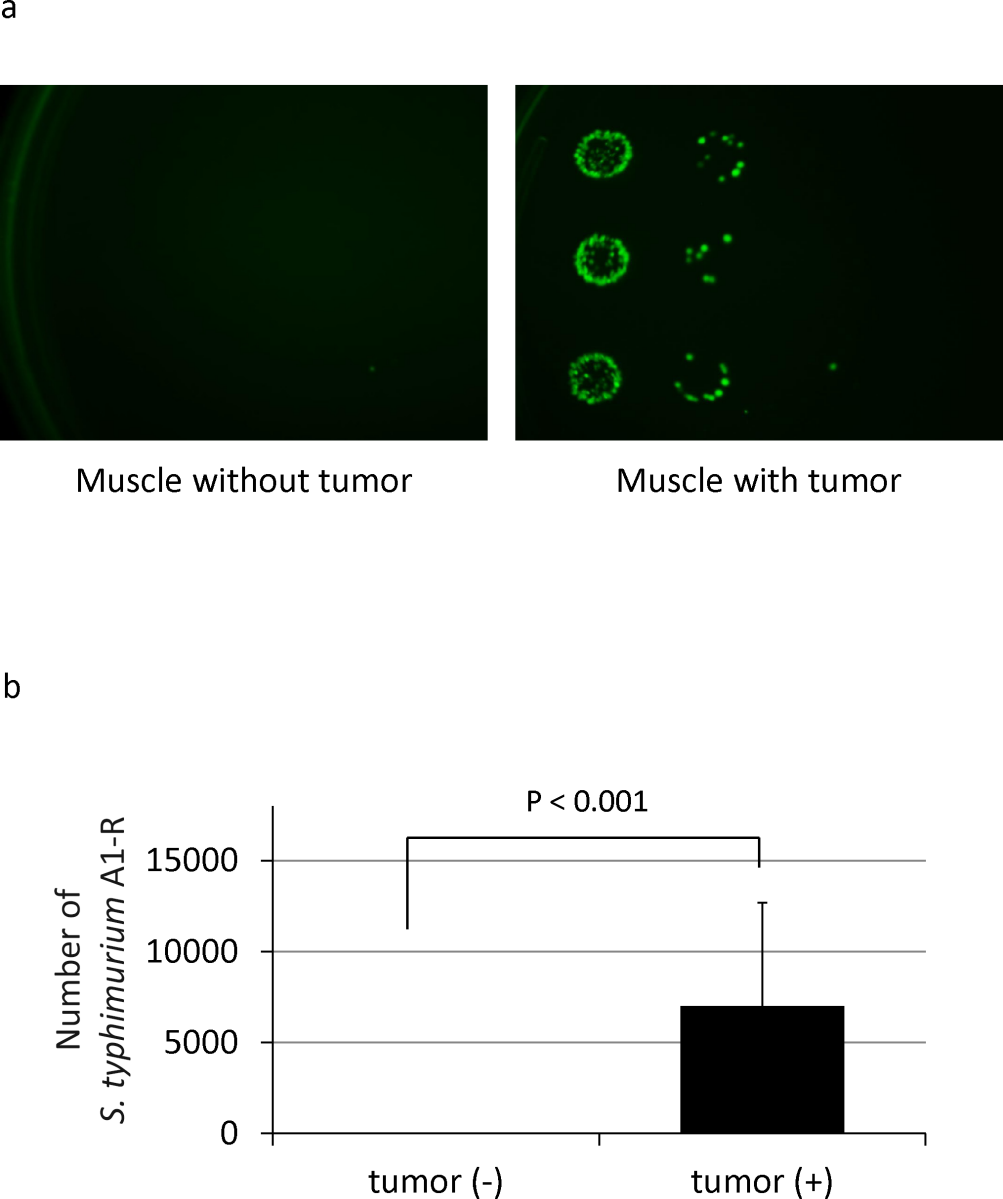
Selective tumor-targeting of *S. typhimurium* A1-R **(a)** *S. typhimurium* A1-R-GFP colonies derived from muscle with or without tumor after *S. typhimurium* A1-R treatment. **(b)** Number of *S. typhimurium* A1-R-GFP in muscle with or without tumor. GFP-expressing *S. typhimurium* A1-R colonies were counted. The colonies were obtained from the tumor tissue after it was minced, diluted with PBS and cultured on ager plates. Normal muscle tissue, from the opposite leg of the mouse treated with *S. typhimurium* A1-R, did not yield observable bacterial colonies after mincing, dilution with PBS and culture on agar plates. The *S. typhimurium* A1-R colonies were observed by their GFP fluorescence. The total number of bacteria was calculated by multiplying the colonies observed by the dilution factor carried out before plating.

HT1080-RFP cells (1 × 10^6^ per mouse) in Matrigel (5 μl) (BD Bioscience, San Jose, CA) were injected into the left femoral muscle. On day 14, *S. typhimuium* A1-R was injected into the tail vein. Three days after *S. typhimuium* A1-R injection, the left femoral muscle with tumor and right femoral muscle without tumor were resected and minced in 1 ml PBS. The PBS containing muscle and/or tumor tissue was diluted and cultured on plates with LB agar. After 24 h culture, *S. typhimuium* A1-R colony formation was observed with the OV100 Small Animal Imaging System (Olympus Corp.) [19] by GFP expression (Fig. 2). These results demonstrated that *S. typhimuium* A1-R selectively targeted and survived only in the tumor tissue.

### Efficacy of *S. typhimuium* A1-R on primary soft tissue sarcoma and spontaneous lung metastases

**Figure 3:**
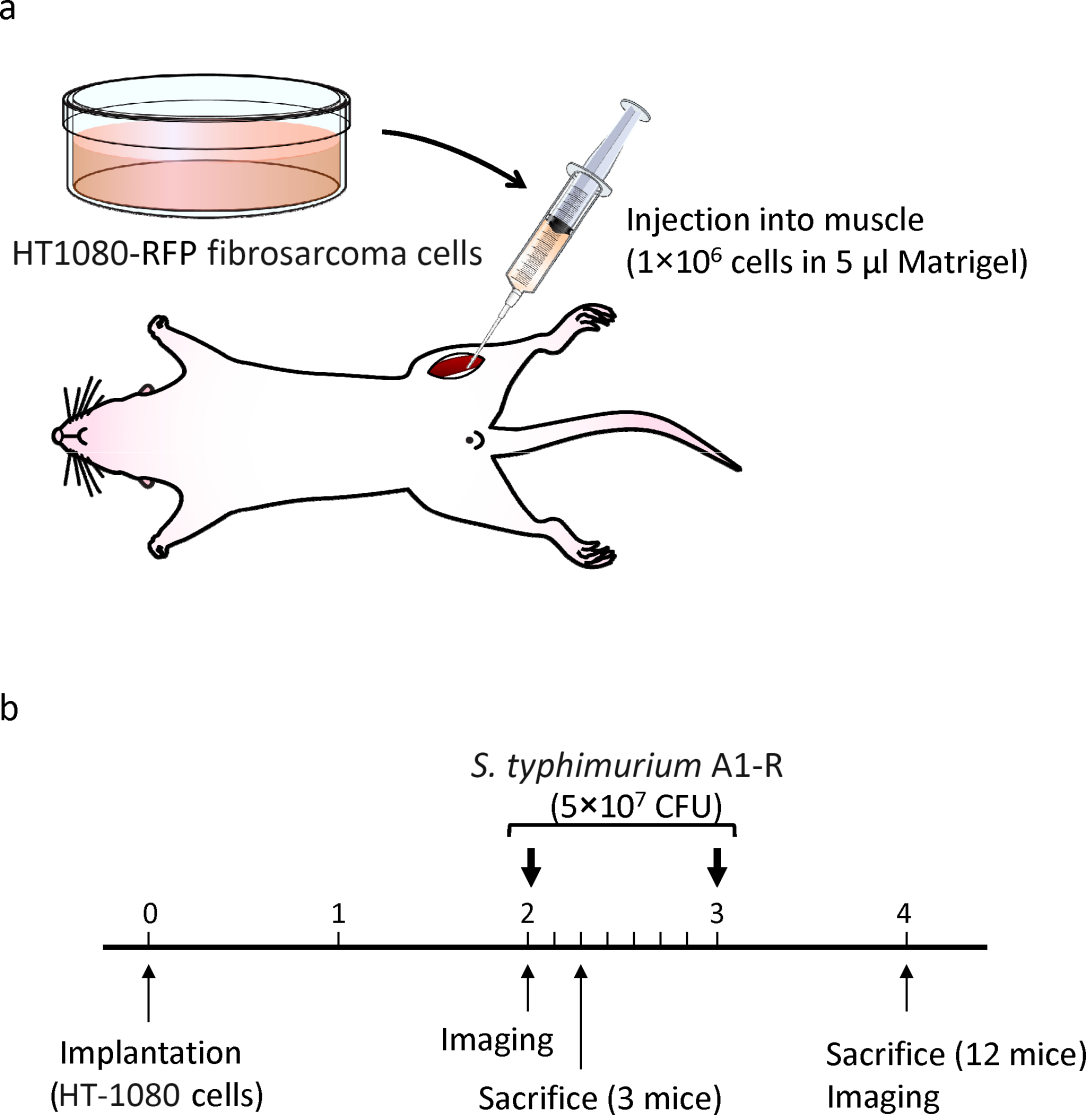
Efficacy determination of *S. typhimurium* A1-R on an orthotopic mouse model of HT-1080 soft tissue fibrosarcoma **(a)** Orthotopic mouse model of HT-1080 soft tissue fibrosarcoma. **(b)** Treatment protocol of *S. typhimurium* A1-R on the orthotopic model of soft-tissue sarcoma.

**Figure 4:**
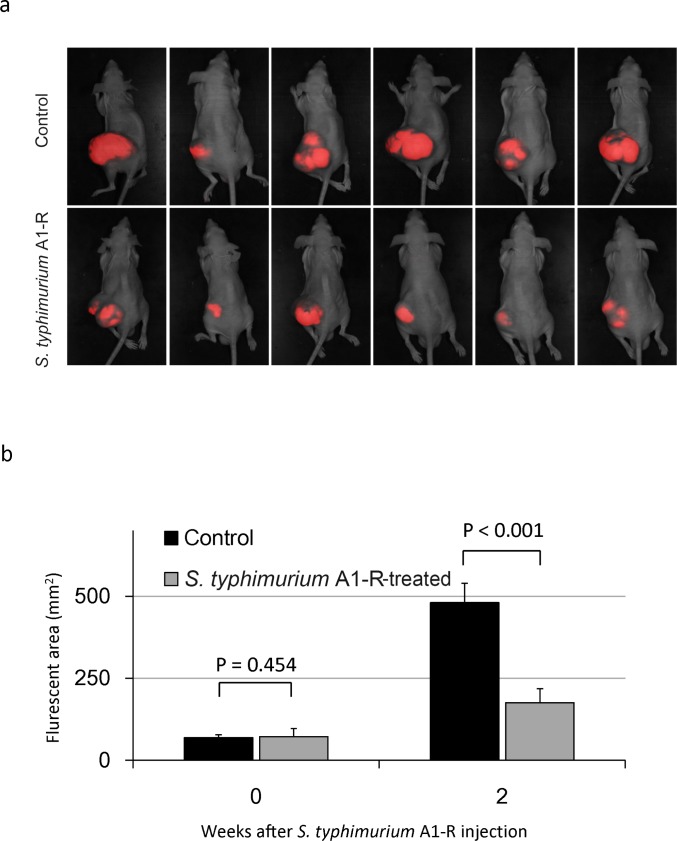

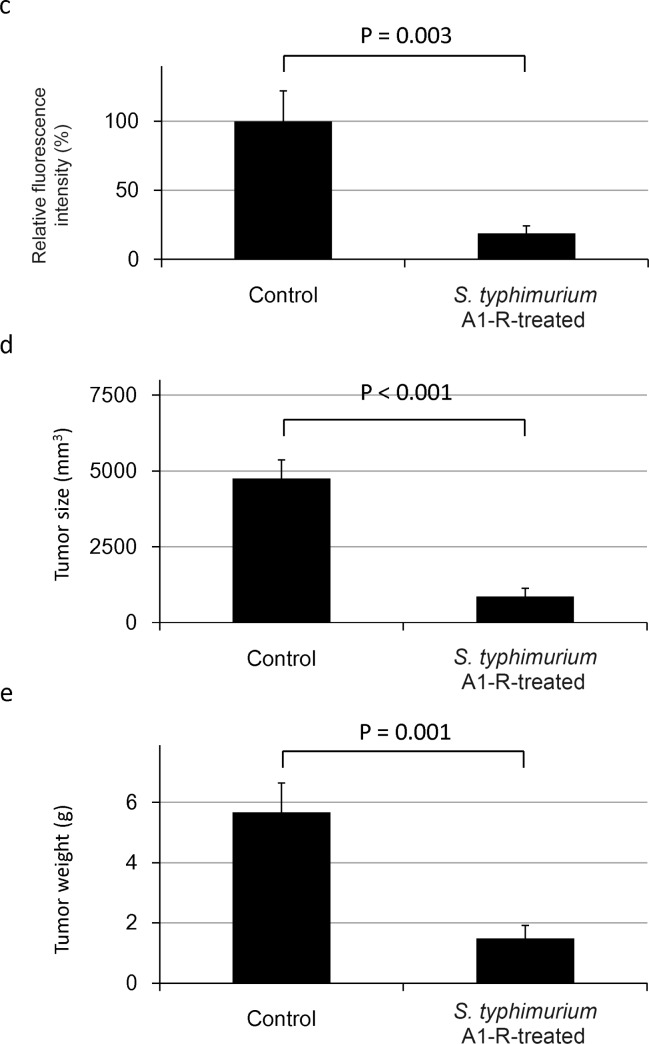
Efficacy of *S. typhimurium* A1-R on an orthotopic mouse model of HT-1080 soft-tissue fibrosarcoma **(a)** *S. typhimurium* A1-R inhibition of soft-tissue sarcoma primary tumor growth on day 28 after implantation. **(b)** Fluorescent areas of soft-tissue sarcoma primary tumor growth with or without *S. typhimurium* A1-R treatment on day 28 after implantation. **(c)** Fluorescence intensity of soft-tissue sarcoma tumors with or without *S. typhimurium* A1-R treatment on day 28 after implantation. **(d)** Tumor size with or without *S. typhimurium* A1-R treatment on day 28 after implantation. **(e)** Tumor weight with or without *S. typhimurium* A1-R treatment on day 28 after implantation.

**Figure 5:**
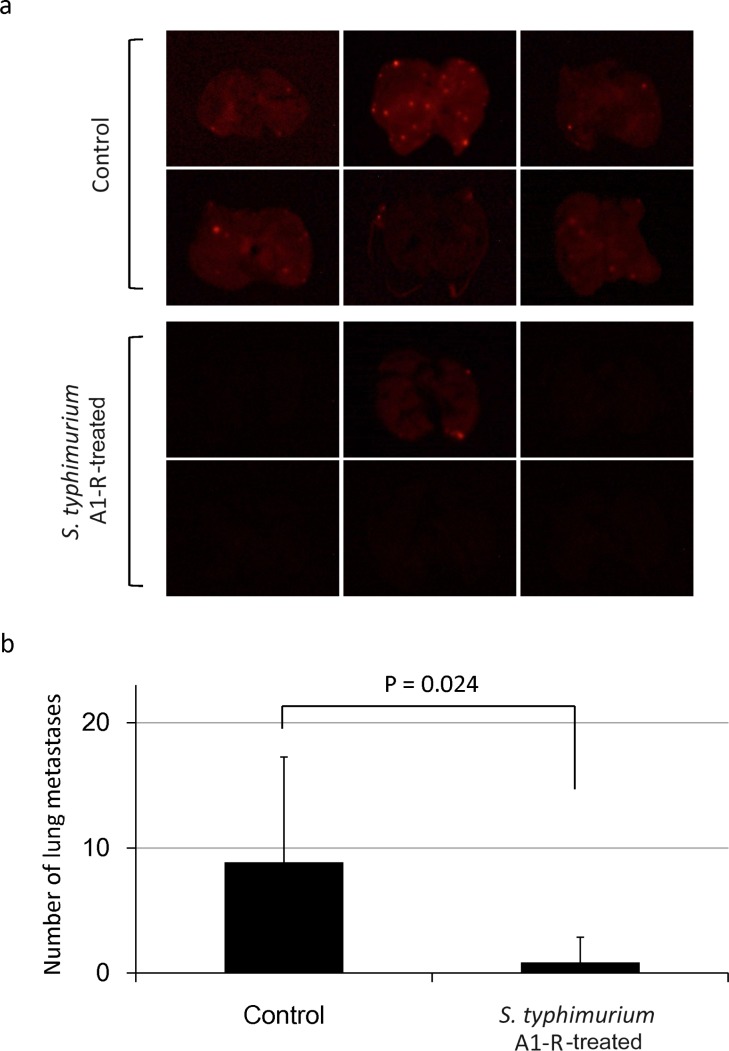
Efficacy of *S. typhimurium* A1-R on spontaneous lung metastasis of HT-1080-RFP soft-tissue sarcoma **(a)** Fluorescence imaging of spontaneous lung metastases from control and *S. typhimurium* A1-R-treated mice. **(b)** Number of lung metastases in control and *S. typhimurium* A1-R-treated mice.

Mice transplanted with HT1080-RFP cells in the leg muscle developed primary soft tissue tumor and lung metastasis (Fig. 3). Fourteen days after tumor injection, the RFP tumor was confirmed by imaging with the iBOX (UVP, LLC, Upland, CA). *S. typhimurium* A1-R was administered on days 14 and 21 after transplantation. On day 28, the fluorescent area of the tumor and lung metastasis was determined with the OV100. The fluorescent area of the primary tumor was 481 ± 59 mm^2^ in the untreated group and 176 ± 42 mm^2^ in the treated group (*P* < 0.001) (Fig. 4a, b). The fluorescence intensity of the treated group was 18.7% of the untreated group (*P* = 0.003) (Fig. 4b). The primary tumor size was 4750 ± 612 mm^3^ in the untreated group and 867 ± 273 mm^3^ in the treated group (*P* < 0.001) (Fig. 4d). The primary tumor weight was 5.7 ± 1.0 g in the untreated group and was 1.5 ± 0.4 g in the treated group (*P* = 0.001) (Fig. 4e). To evaluate the efficacy of *S. typhimurium* A1-R on spontaneous lung metastases, the lungs were excised and the metastases on the surface were counted with the OV100 (Fig. 5a). The number of metastasis was 8.8 ± 3.4 per mouse in the untreated group and 0.8 ± 0.8 in the treated group (*P* = 0.024) (Fig. 5b).

### Efficacy of *S. typhimurium* A1-R on fibrosarcoma experimental lung metastasis

**Figure 6:**
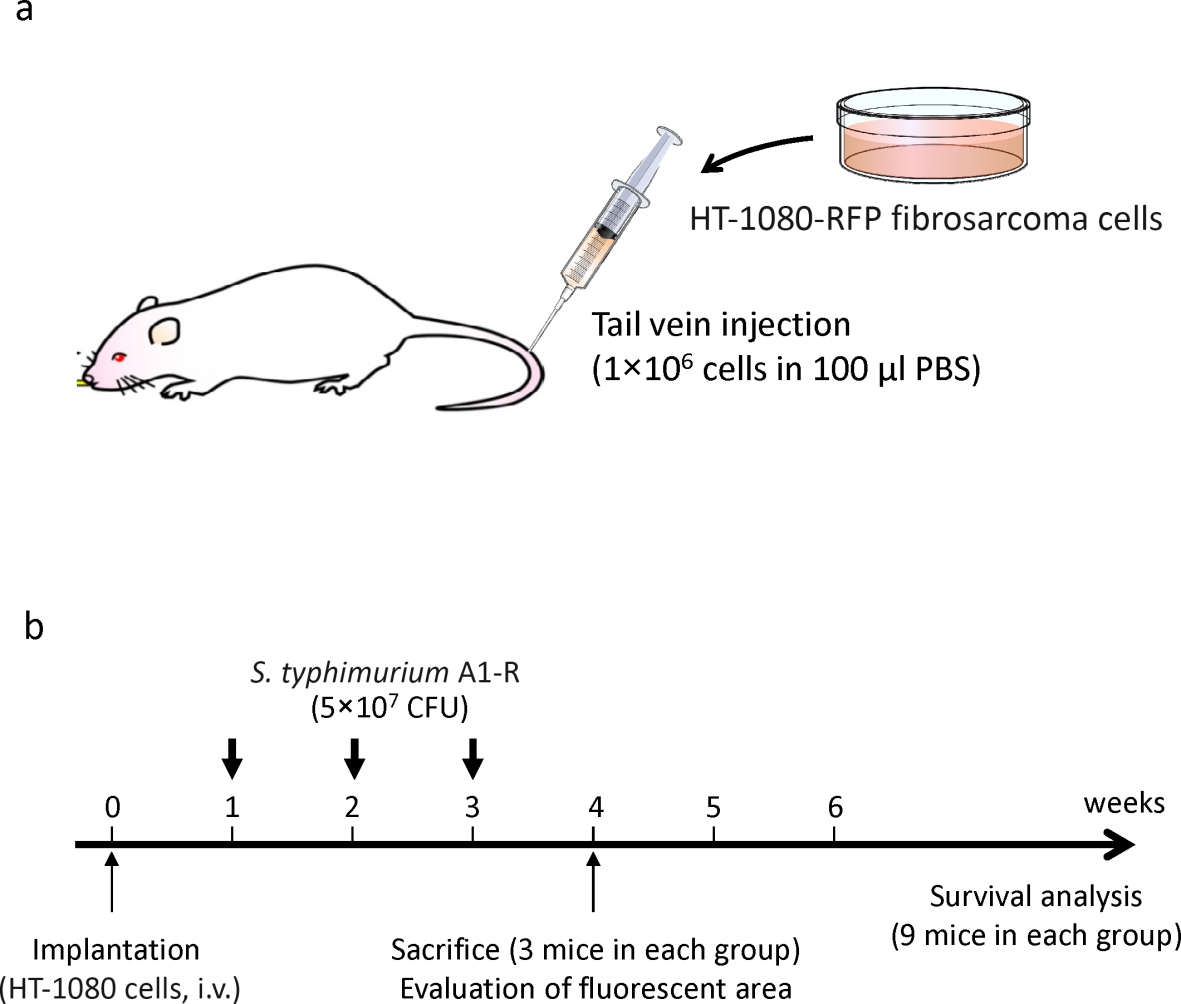

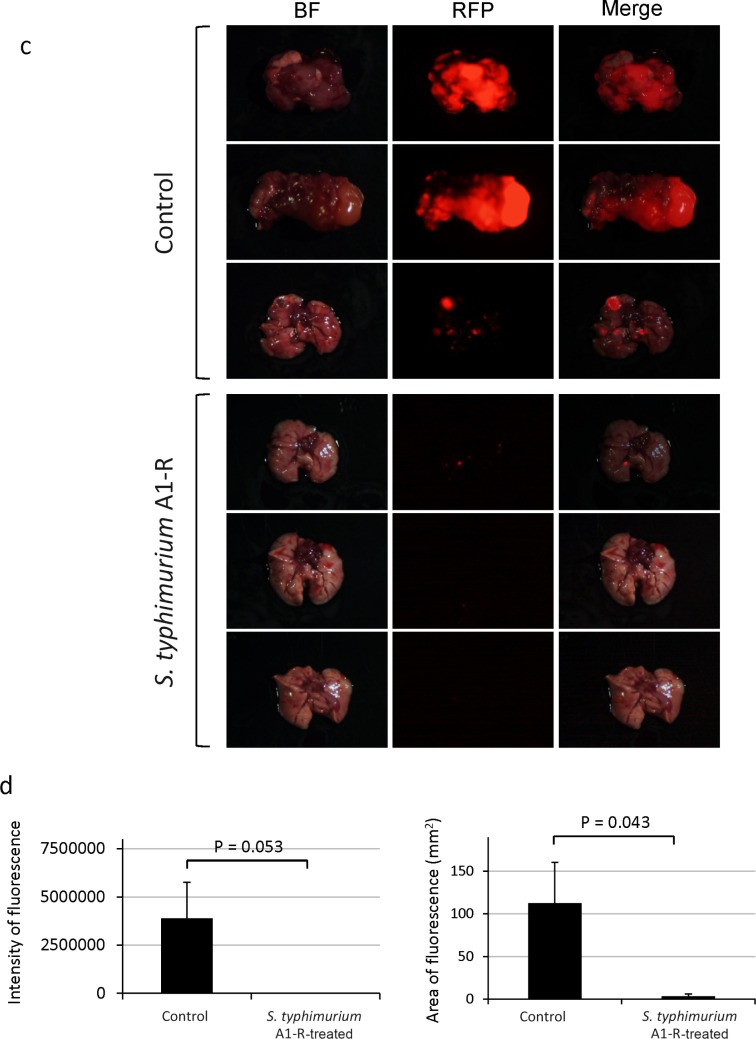

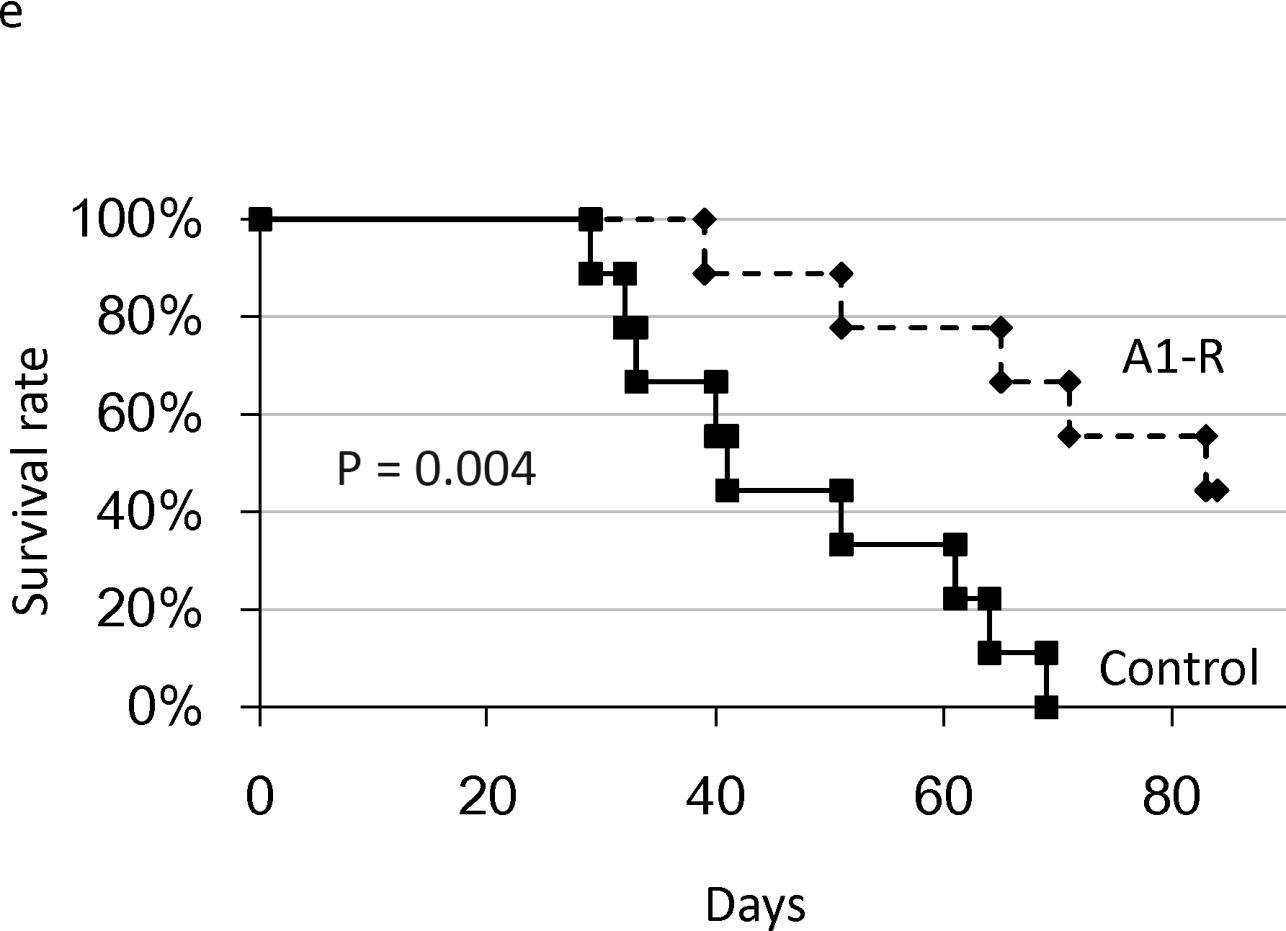
Efficacy of *S. typhimurium* A1-R on experimental lung metastasis of fibrosarcoma **(a)** Mouse model of soft-tissue sarcoma experimental lung metastasis. **(b)** Treatment protocol. **(c)** Fluorescence imaging of lung metastases with and without *S. typhimurium* A1-R treatment. **(d)** Fluorescence intensity and area of soft-tissue sarcoma lung metastases. BF = brightfield. **(e)** Kaplan-Meier survival curve of mice with soft-tissue sarcoma treated with *S. typhimurium* A1-R compared to control untreated mice.

HT1080-RFP cells (1 × 10^6^ cells in 100 μl PBS) were injected into the tail vein of 24 nude mice (day 0) (Fig. 6a). On days 7, 14, and 21, *S. typhimurium* A1-R (5 × 10^7^ CFU per mouse) was injected into the tail vein (Fig. 6b). On day 28, 6 mice (3 mice of each group) were sacrificed and the lungs were imaged to determine the efficacy of bacterial therapy on lung metastases. Fluorescence imaging demonstrated that *S. typhimurium* A1-R strongly inhibited lung metastases (Fig. 6c). The mean fluorescence intensity of lung metastases of the control mice and *S. typhimurium* A1-R treated mice was 3.9 × 10^6^ and 4.8 × 10^3^, respectively, an almost 1,000-fold decrease in the treated mice (*P* = 0.053; Fig. 6d). The fluorescence area of the lung metastases of control mice and *S. typhimurium* A1-R-treated mice was 112.4 ± 48.1 mm^2^ and 3.3 ± 2.7 mm^2^, respectively (*P* = 0.043; Fig. 6d). Kaplan–Meier analysis with the log rank test demonstrated that *S. typhimurium* A1-R significantly improved the survival of the treated mice (*P* = 0.004; Fig. 6e).

In the present study, two models of soft-tissue lung metastasis comprising spontaneous metastasis and experimental metastasis were used to assess the efficacy of *S. typhimurium* A1-R. In the orthotopic spontaneous metastasis model of soft tissue sarcoma, *S. typhimurium* A1-R significantly inhibited primary tumor growth and spontaneous lung metastases. The experimental lung metastasis model was used to assess the direct effect of *S. typhimurium* A1-R on lung colonization. *S. typhimurium* A1-R strongly inhibited lung colonization. Furthermore, *S. typhimurium* A1-R significantly improved the survival of the mice.

Thus, *S. typhimurium* A1-R directly inhibits primary tumor growth and metastasis of soft-tissue sarcoma. The present study suggests that *S. typhimurium* A1-R therapy has superior potential for the systemic treatment of soft tissue sarcoma metastasis than *C. novyi* (NT) that appears to be limited to i.t, injection [17]. The comparison of the anti-tumor and anti-metastatic efficacy of the two types of bacteria will require future clinical trials.

## MATERIALS AND METHODS

### Preparation of *S. typhimurium* A1-R

GFP-expressing *Salmonella typhimurium* A1-R (AntiCancer Inc., San Diego, CA, USA) were grown overnight in LB medium (Fisher Sci., Hanover Park, IL, USA) and then diluted 1:10 in LB medium. Bacteria were harvested at late-log phase, washed with PBS, and then diluted in PBS [3, 5]. Bacteria were then ready for *in vitro* or *in vivo* experiments.

### Efficacy of *S. typhimurium* A1-R on HT1080 fibrosarcoma cells *in vitro*

To evaluate the ability of *S. typhimurium* A1-R to kill human fibrosarcoma cells *in vitro*, the interaction between *S. typhimurium* A1-R expressing GFP and HT1080-RFP cells was observed with the Fluoview FV1000 confocal microscope (Olympus Corp., Tokyo, Japan). HT1080-RFP cells were cultured in 35 mm dishes for 24 h. *S. typhimurium* A1-R bacteria were grown in LB and added to the fibrosarcoma cells (1 × 10^8^ CFU/dish or 1 × 10^9^ CFU/dish). After 1 h incubation at 37°C, the cells were rinsed and cultured in medium containing gentamycin sulfate (100 μg/ml) to kill external but not internal bacteria [3].

### Animals

Athymic (*nu*/nu) nude mice (AntiCancer, Inc. San Diego, CA) were used in this study. Mice were kept in a barrier facility under high efficiency particulate air (HEPA) filtration. Mice were fed with autoclaved laboratory rodent diet. All animal studies were conducted in accordance with the principles and procedures outlined in the National Institutes of Health Guide for the Care and Use of Laboratory Animals under Assurance no. A3873-1.

### Orthotopic mouse model of soft tissue sarcoma

Six-week old female nude mice were anesthetized by a ketamine mixture (10 μl ketamine, HCL, 7.6 μl xylazine, 2.4 μl acepromazine maleate and 10 μl H_2_O) via s.c. injection. The leg was sterilized with alcohol and an approximately 2 mm midline skin incision was made just above the knee joint to expose the quadriceps femoris muscle. HT1080-RFP cells (1 × 10^6^ per mouse) in Matrigel (5 μl per mouse) (BD Bioscience, San Jose, CA) were injected into the muscle with a 0.5 ml 28 G latex-free insulin syringe (TYCO Health Group LP, Mansfield, MA). The skin was closed with a 6-0 suture. On day 14 and 21, *S. typhymurium* (5 × 10^7^ CFU per mouse) was injected into the tail vein. On day-28, the mice were sacrificed and fluorescence imaging was performed to determine the efficacy of bacterial therapy for both primary tumors and lung metastases. The size of the primary tumors (fluorescent area [mm^2^]) was measured with the iBox Imaging System (UVP LLC, Upland, CA, USA). The lung tumor was excised and the metastases on the surface were imaged and counted with the OV100 Small Animal Imaging System (Olympus Corp., Tokyo, Japan).

### Experimental lung metastasis model of soft tissue sarcoma

Six-week-old female nude mice were used. To obtain experimental lung metastasis, HT1080-RFP cells (1 × 10^6^ cells in 100 μl PBS) were injected into the tail vein of 24 nude mice (day 0). On days 7, 14, and 21, *S. typhimurium* A1-R (5 × 10^7^ CFU) was injected in the tail vein. Twelve mice were treated with bacteria and 12 mice were used as untreated control. On day 28, 6 mice (3 mice each group) were sacrificed and the lungs were imaged to observe lung metastases and to determine the efficacy of bacterial therapy. Lung metastases were observed and the fluorescent areas were recorded using the OV100. Additionally, 18 mice comprising 9 control mice and 9 *S. typhimurium* A1-R-treated mice were observed for survival analysis.

### Statistical analysis

Data showing comparisons between two groups were assessed using the Student's *t*-test. Kaplan–Meier analysis with the log-rank test was used to determine survival difference between treatment groups. Differences were considered significant when p ≤ 0.05. The experimental data are expressed as the mean ± SE.

## References

[1] Takeuchi A, Tsuchiya H, Yamamoto N, Hayashi K, Yamauchi K, Kawahara M, Miyamoto K, Tomita K (2007). Caffeine-potentiated chemotherapy for patients with high-grade soft tissue sarcoma: long-term clinical outcome. Anticancer Res.

[2] Nystrom LM, Reimer NB, Reith JD, Dang L, Zlotecki RA, Scarborough MT, Gibbs CP (2013). Multidisciplinary management of soft tissue sarcoma. Scientific World Journal.

[3] Zhao M, Yang M, Li XM, Jiang P, Baranov E, Li S, Xu M, Penman S, Hoffman RM (2005). Tumor-targeting bacterial therapy with amino acid auxotrophs of GFP-expressing *Salmonella typhimurium*. Proc Natl Acad Sci USA.

[4] Zhao M, Geller J, Ma H, Yang M, Penman S, Hoffman RM (2007). Monotherapy with a tumor-targeting mutant of *Salmonella typhimurium* cures orthotopic metastatic mouse models of human prostate cancer. Proc Natl Acad Sci USA.

[5] Zhao M, Yang M, Ma H, Li X, Tan X, Li S, Yang Z, Hoffman RM (2006). Targeted therapy with a *Salmonella typhimurium* leucine-arginine auxotroph cures orthotopic human breast tumors in nude mice. Cancer Res.

[6] Uchugonova A, Zhao M, Zhang Y, Weinigel M, König K, Hoffman RM (2012). Cancer-cell killing by engineered Salmonella imaged by multiphoton tomography in live mice. Anticancer Res.

[7] Liu F, Zhang L, Hoffman RM, Zhao M (2010). Vessel destruction by tumor-targeting *Salmonella typhimurium* A1-R is enhanced by high tumor vascularity. Cell Cycle.

[8] Nagakura C, Hayashi K, Zhao M, Yamauchi K, Yamamoto N, Tsuchiya H, Tomita K, Bouvet M, Hoffman RM (2009). Efficacy of a genetically-modified *Salmonella typhimurium* in an orthotopic human pancreatic cancer in nude mice. Anticancer Res.

[9] Yam C, Zhao M, Hayashi K, Ma H, Kishimoto H, McElroy M, Bouvet M, Hoffman RM (2010). Monotherapy with a tumor-targeting mutant of S. typhimurium inhibits liver metastasis in a mouse model of pancreatic cancer. J Surg Res.

[10] Hiroshima Y, Zhao M, Zhang Y, Maawy A, Hassanein MK, Uehara F, Miwa S, Yano S, Momiyama M, Suetsugu A, Chishima T, Tanaka K, Bouvet M, Endo I, Hoffman RM (2013). Comparison of efficacy of *Salmonella typhimurium* A1-R and chemotherapy on stem-like and non-stem human pancreatic cancer cells. Cell Cycle.

[11] Hiroshima Y, Zhao M, Maawy A, Zhang Y, Katz MH, Fleming JB, Uehara F, Miwa S, Yano S, Momiyama M, Suetsugu A, Chishima T, Tanaka K, Bouvet M, Endo I, Hoffman RM (2014). Efficacy of *Salmonella typhimurium* A1-R versus chemotherapy on a pancreatic cancer patient-derived orthotopic xenograft (PDOX). J Cell Biochem.

[12] Hayashi K, Zhao M, Yamauchi K, Yamamoto N, Tsuchiya H, Tomita K, Hoffman RM (2009). Cancer metastasis directly eradicated by targeted therapy with a modified *Salmonella typhimurium*. J Cell Biochem.

[13] Hayashi K, Zhao M, Yamauchi K, Yamamoto N, Tsuchiya H, Tomita K, Kishimoto H, Bouvet M, Hoffman RM (2009). Systemic targeting of primary bone tumor and lung metastasis of high-grade osteosarcoma in nude mice with a tumor-selective strain of *Salmonella typhimurium*. Cell Cycle.

[14] Kimura H, Zhang L, Zhao M, Hayashi K, Tsuchiya H, Tomita K, Bouvet M, Wessels J, Hoffman RM (2010). Targeted therapy of spinal cord glioma with a genetically-modified *Salmonella typhimurium*. Cell Proliferation.

[15] Momiyama M, Zhao M, Kimura H, Tran B, Chishima T, Bouvet M, Endo I, Hoffman RM (2012). Inhibition and eradication of human glioma with tumor-targeting *Salmonella typhimurium* in an orthotopic nude-mouse model. Cell Cycle.

[16] Miwa S, Yano S, Zhang Y, Matsumoto Y, Uehara F, Yamamoto M, Hiroshima Y, Kimura H, Hayashi K, Yamamoto N, Bouvet M, Tsuchiya H, Hoffman RM, Zhao M (2014). Tumor-targeting *Salmonella typhimurium* A1-R prevents experimental human breast cancer bone metastasis in nude mice. Oncotarget.

[17] Roberts NJ, Zhang L, Janku F, Collins A, Bai RY, Staedtke V, Rusk AW, Tung D, Miller M, Roix J, Khanna KV, Murthy R, Benjamin RS, Helgason T, Szvalb AD, Bird JE, Roy-Chowdhuri S, Zhang HH, Qiao Y, Karim B, McDaniel J, Elpiner A, Sahora A, Lachowicz J, Phillips B, Turner A, Klein MK, Post G, Diaz LA, Riggins GJ, Papadopoulos N, Kinzler KW, Vogelstein B, Bettegowda C, Huso DL, Varterasian M, Saha S, Zhou S (2014). Intratumoral injection of Clostridium novyi-NT spores induces antitumor responses. Science Transl Med.

[18] Yazawa K, Fujimori M, Nakamura T, Sasaki T, Amano J, Kano Y, Taniguchi S (2001). Bifidobacterium longum as a delivery system for gene therapy of chemically induced rat mammary tumors. Breast Cancer Res Treat.

[19] Yamauchi K, Yang M, Jiang P, Xu M, Yamamoto N, Tsuchiya H, Tomita K, Moossa AR, Bouvet M, Hoffman RM (2006). Development of real-time subcellular dynamic multicolor imaging of cancer-cell trafficking in live mice with a variable-magnification whole-mouse imaging system. Cancer Res.

